# Correlates of preclinical cardiovascular disease in Indigenous and Non-Indigenous Australians: a case control study

**DOI:** 10.1186/1476-7120-6-36

**Published:** 2008-07-16

**Authors:** Brian A Haluska, Lionel Chan, Leanne Jeffriess, A Andrew Shaw, Joanne Shaw, Thomas H Marwick

**Affiliations:** 1 School of Medicine, University of Queensland, Brisbane, Australia

## Abstract

**Background:**

The high frequency of premature death from cardiovascular disease in indigenous Australians is often attributed to the high prevalence of risk factors, especially type II diabetes mellitus (DM). We evaluated the relationship of ethnicity to atherosclerotic burden, as evidenced by carotid intima-media thickness (IMT), independent of risk factor status.

**Methods:**

We studied 227 subjects (147 men; 50 ± 13 y): 119 indigenous subjects with (IDM, n = 54), and without DM (InDM, n = 65), 108 Caucasian subjects with (CDM, n = 52), and without DM (CnDM, n = 56). IMT was measured according to standard methods and compared with clinical data and cardiovascular risk factors.

**Results:**

In subjects both with and without DM, IMT was significantly greater in indigenous subjects. There were no significant differences in gender, body mass index (BMI), systolic blood pressure (SBP), or diastolic blood pressure (DBP) between any of the groups, and subjects with DM showed no difference in plasma HbA1c. Cardiovascular risk factors were significantly more prevalent in indigenous subjects. Nonetheless, ethnicity (β = -0.34; p < 0.0001), age (β = 0.48; p < 0.0001), and smoking (β = 0.13; p < 0.007) were independent predictors of IMT in multiple linear regression models.

**Conclusion:**

Ethnicity appears to be an independent correlate of preclinical cardiovascular disease, even after correction for the high prevalence of cardiovascular risk factors in indigenous Australians. Standard approaches to control currently known risk factors are vital to reduce the burden of cardiovascular disease, but in themselves may be insufficient to fully address the high prevalence in this population.

## Background

In 2001 there were approximately 460,000 indigenous people in Australia, accounting for 2.4% of the population. However persons greater than 40 years old account for proportionately fewer indigenous people, reflecting the fact that indigenous people are much more likely to die before they are old than the general Australian public: men at 56 years; women at 63 years [1-9]. In addition, death rates are estimated to be four times higher in indigenous than in non-indigenous Australians.

In 2002 the leading cause of death in indigenous people was cardiovascular disease (CVD), responsible for 1/3 of all deaths, followed by ischemic heart disease (16%) and stroke (9%) [1-8]. Of indigenous Australians aged 35–44 years, 16% reported a cardiovascular condition, with the rate increasing to 31% for those aged 45 to 54 years, and to 47% for those aged 55 years and over. The prevalence of cardiovascular disease is greater in remote areas. Coronary heart disease is 3–4 times higher for males and females than in non-indigenous people [10,11]. Indigenous people are much more likely to die of CVD than non-indigenous people at any age, especially in younger age groups – the death rate among 25–54 year olds was 10 times higher than other Australians.

Atherosclerotic risk factors are more prevalent in the indigenous community; hypertension is the most common risk factor. Diabetes mellitus (DM) is 2–4 times more prevalent in indigenous than in non-indigenous people, with the onset of DM at a younger age [10,11], and a higher reported mortality (7.6% vs. 2.4%) in indigenous patients with DM than in their Caucasian counterparts. Chan et al [12] have reported that traditional cardiovascular risk factors are associated with increased atherosclerotic burden (measured by carotid intima-media thickness [IMT]) in indigenous subjects. In this study we sought whether this preclinical cardiovascular disease was related to ethnicity (which is a marker of race and a number of socio-economic factors) independent of differences in risk factors.

## Methods

### Patient selection

We studied 227 patients (147 men; 50 ± 13 y) from North Stradbroke Island, Brisbane, and the Redland Bay area in southeast Queensland, Australia: 54 indigenous patients with DM (IDM), 65 indigenous patients without DM (InDM), 52 Caucasian patients with DM (CDM) and 56 Caucasian patients with no DM (CnDM). Clinical characteristics (age, gender, glycosylated hemoglobin [HbA1c], BMI, risk factors (smoking, HTN, increased total cholesterol (TC)), resting blood pressure, and results for vascular structure (IMT) were compared between indigenous and non-indigenous groups and between the patients with and without DM. Blood pressure was taken in the sitting position in the right arm in all patients using as standard sphygmomanometer after they had been allowed to rest for approximately 5–10 minutes. Three measurements were taken and the second and third were averaged and reported. Hypertension (HTN) was defined as resting blood pressure > 135/90 or on medication for HTN or by patient history. Increased TC was defined as a fasting total cholesterol of >5.5 mmol/L or on medication.

### Carotid imaging

Carotid IMT was measured from anterior, lateral and posterior longitudinal scans of the left and right extra-cranial carotid arteries, using a standard ultrasound system and a 12 MHz imaging probe (ATL HDI5000, Philips Medical Systems, Bothell WA). Off-line measurement of the common carotid artery was performed using automated software (IMT v3.17 plug-in, HDILab v1.91c, Philips, Bothell, WA) which uses a maximum slope edge detection method to calculate the mean and standard deviation for the IMT thickness in the region of interest box. If there was plaque in the carotid artery, care was taken not to include the plaque in the IMT measurement. Because of noise and gain dependency in the near field, only the posterior wall measurements were recorded, and the average measurements of the anterior, lateral and posterior walls from both right and left carotid arteries was used for comparison between groups. Intra-observer variation and coefficient of variance from this institution for IMT are 0.01 ± 0.04 mm (5%).

### Statistical analysis

Independent samples t-tests and ANOVA with Bonferoni's post-hoc test were used to determine difference in the means between the groups of patients. Subgroup analysis was performed by decade of age for age corrected IMT, and linear regression models were then used to determine the correlates of IMT in the different groups.

## Results

### Clinical characteristics

The clinical features, blood pressure and cardiovascular risk factors for the indigenous and non-indigenous groups with and without DM are shown in Table 1. There were significant differences in age within the indigenous and non-indigenous groups but no differences in age between the patients with and without DM within each group. The IDM did have more risk factors than all of the other groups; however they were only significantly different from the CnDM group. There was no significant difference in gender, body mass index (BMI) or diastolic blood pressure (DBP) in any of the groups, and there was also no significant difference in plasma HbA1c between the indigenous and non-indigenous groups with DM.

**Table 1:** Clinical data, blood pressure and cardiovascular risk factors by group for the patient cohort.

	CnDM 56	CDM 52	InDM 65	IDM 54
Age (yr)	41 ± 7	56 ± 11	46 ± 13	57 ± 13
Male	35 (62%)	33 (63%)	35 (54%)	35 (65%)
HbA1c (%)	N/A	7.4 ± 1.7	5.7 ± .60	7.8 ± 1.8
BMI (kg/m^2^)	31 ± 5	31 ± 4	30 ± 8	31 ± 6
SBP (mmHg)	120 ± 12	125 ± 15	126 ± 17	132 ± 17**
DBP (mmHg)	78 ± 8	80 ± 9	78 ± 12	78 ± 11
Smoking	1 (2%)	20 (38%)	34 (52%)	50 (92%)**
HTN	2 (3.5%)	22 (42%)	39 (60%)	40 (74%)**
↑TC	3 (5%)	26 (50%)	30 (46%)	39 (72%)**

**p < 0.0001 versus CnDM				

Results are in mean ± SD or number (%). BMI – body mass index; SBP – systolic blood pressure; DBP diastolic blood pressure; HTN hypertension; ↑TC – increased total cholesterol; CnDM – Caucasian non-diabetic; CDM – Caucasian diabetic; InDM – Indigenous non-diabetic; IDM – Indigenous diabetic.

### Carotid intima-media thickness

**Figure 1:**
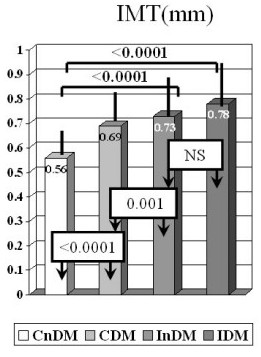
Bar graphs representing mean intimal-medial thickness (IMT) for the four groups of patients; CnDM-Caucasian non-diabetics, CDM-Caucasian diabetics, InDM-indigenous non-diabetics, IDM-indigenous diabetics.

The results for IMT showed significant differences between and within most groups (Figure 1). All of the groups had significantly greater IMT compared to the CnDM group and both indigenous groups had significantly greater IMT compared with the non-indigenous groups, including the CDM group. There were no significant differences in IMT between the IDM and InDM groups and both were in the abnormal range.

### Age corrected IMT and subgroup analysis

**Figure 2:**
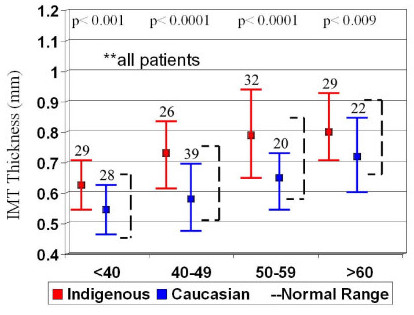
Subgroup analysis of mean IMT by decade of age in the entire patient cohort with normal values (broken lines)

**Figure 3:**
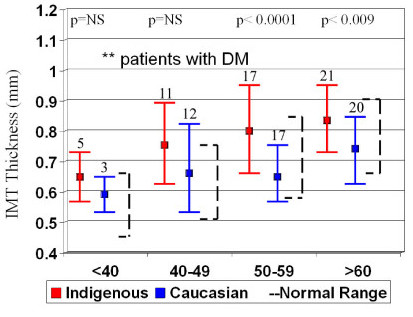
Subgroup analysis of mean IMT by decade of age in patients with diabetes (DM) with normal values (broken lines)

**Figure 4:**
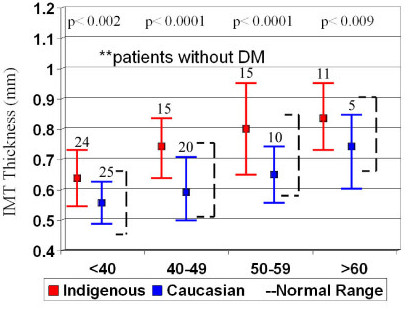
Subgroup analysis of mean IMT by decade of age in patients without diabetes (DM) with normal values (broken lines)

IMT increases with age, so the patient cohort was analysed by decade of age to determine age-related differences. For the entire patient cohort the Caucasian groups were well within the normal range of IMT for age and significantly lower than the Indigenous groups (Figure 2). In addition, the indigenous IMT was above the normal ranges for each decade, even in patients <40 years of age. The same was seen when the groups were divided into patients with DM (Figure 3) and those without DM (Figure 4), however there were only significant differences in IMT in patients >50 years old with DM.

### Determinants of preclinical atherosclerosis

The independent correlates of IMT were sought in a linear regression model. For the entire patient cohort the independent correlates were age (β = 0.46; p < 0.0001), ethnicity (β = -0.29; p < 0.0001), and the incidence of cigarette smoking (β = 0.19; p < 0.003), with a good model fit (R = 0.69; p < 0.0001). The same regression models were then used for the patients with and without DM. For patients with DM the independent correlates were age (β = 0.30; p < 0.002), ethnicity (β = 0.32; p < 0.001), cigarette smoking (β = 0.29; p < 0.003), and plasma HbA1c (β = 0.22; p < 0.02) (Model R = 0.59; p < 0.0001). In patients without DM they were age (β = 0.47; p < 0.0001), and ethnicity (β = -0.48; p < 0.0001) (Model R = 0.76; p < .0001).

## Discussion

The results of this study suggest that ethnic background (or its milieu) plays a role in addition to risk factors in the development of early cardiovascular disease in indigenous Australians both with and without DM. While the Caucasian patients with DM had significantly higher IMT than those without, in the age-adjusted analysis, both groups of Caucasian patients had significantly lower IMT than both of the indigenous groups.

### Use of IMT as a marker of atherosclerotic burden

Intima-media thickness is a marker for early atherosclerosis and may be used to serially follow patients with this disease [15]. Although the amount of atheroma formation in different arteries varies, atheroma formation is a diffuse process, with a good correlation between brachial, carotid and coronary arterial lesions by post mortem [16]. Carotid IMT reflects atherosclerotic burden and increases in IMT of 0.1 mm can increase the risk of stroke or myocardial infarction by as much as 11% [16,17].

While there are much data about risk factors and cardiovascular disease in indigenous Australians, little has been published regarding the presence of preclinical disease [12,18,19]. The literature has generally concluded that cardiovascular risk factors are responsible for the high incidence of established and preclinical atherosclerosis in indigenous Australians. The role of ethnicity has not been addressed [12,18,19], although it may attest to not only genetic susceptibility but also education and socio-economic factors that may influence food behaviour and medical treatment, among many possible contributors. The identification of a risk factor-independent association between ethnicity and atherosclerotic burden would imply that the difference in outcome between indigenous and non-indigenous Australians is unlikely to be reversed by risk factor interventions alone.

### Determinants of atherosclerosis

The Framingham risk score, based on age, gender, systolic or diastolic blood pressure, total cholesterol, smoking status and incidence of DM [20], has been used widely in the prediction of risk. Although the predictive value of this score among different populations has been debated [21-25], several streams of data suggest that it may be reliable. The InterHeart study [26], a large case-control study in over 15,000 cases and over 14,000 controls involving 52 countries concluded that nine easily measurable and modifiable simple risk factors accounted for >90% of the risk of an initial acute myocardial infarction. A subgroup analysis showed excess risk due to abdominal obesity, tobacco use, abnormal lipids, a history of hypertension and diabetes in several ethnic groups, and these risks appear to be important risk factors in every region of the world. Other multivariate risk scores developed from Australian data in Dubbo (New South Wales) and Busselton (Western Australia) [21-24] used the same cardiovascular risk factors used in the Framingham study. In these scores, the estimated risk reported for both men and women between 40 and 80 years of age were not very different to those reported in Framingham [21-24].

Ravikumar et al compared IMT and arterial function in DM and non-DM patients [27] and concluded that increased arterial stiffness and decreased function were present in DM patients compared to matched non-DM patients. While IMT was significantly higher in DM patients, IMT was inversely correlated with arterial stiffness and function in the entire cohort and in non-DM but not in the patients with DM. The data presented here is from indigenous and Caucasian patients with and without DM. The indigenous DM group had the highest incidence of risk factors (smoking, HTN, hyperlipidemia) which was significantly greater than all of the other groups. However there were no other differences in any of those risk factors between the other groups except for incidence of cigarette smoking. And in the age-adjusted analysis of IMT both indigenous groups had abnormal IMT for each decade of age. This suggests other factors in addition to cardiovascular risk factors which may be responsible for development of preclinical atherosclerosis. The independent correlation of ethnicity with increased IMT in all of the linear regression models may be a marker of these other characteristics.

### Other contributors to cardiovascular disease

Data from the British Regional Heart Study have suggested that social class is highly correlated with the incidence of non-fatal myocardial infarction and ischemic heart disease [28]. The high prevalence of socio-economic disadvantage among indigenous Australians [7] may be an explanation of the propensity for preclinical disease over and above that expected from their risk factor status. In similar data from over 15,000 patients in the Atherosclerosis Risk in Communities Study, progressively lower social class, education and income were associated with progressively increased IMT thickness [29]. In that study, the association between education and increased IMT was greater in Caucasians than in African-Americans although they did not specifically address racial and gender differences in cardiovascular disease [29], but even after adjustment was done for risk factors, the associations of lower occupational group and clinical coronary disease persisted [29]. Emotional stress and depression and other psychosocial factors may be more prevalent in patients of lower socioeconomic class and may also play a role in the increased risk of cardiovascular disease [26].

Finally, a genetic contribution to cardiovascular disease has been proposed in the Pima Indians of Arizona and Mexico [25,30,31] Despite the highest incidence of type-II DM and obesity in the world, these groups have a relatively low rate of ischemic heart disease and cardiovascular disease [25]. Diet has also been suggested as a contributor to development of disease and in the same population is was shown that living a "traditional lifestyle" of low saturated fats and higher vegetable intake along with increased physical activity, there was a much lower incidence of type II DM and obesity.

## Conclusion

Despite a high incidence of risk factors in indigenous Australians both with and without DM, ethnicity (and various other risk factors for which it is a marker) appears to be an independent predictor of preclinical cardiovascular disease. Although aggressive risk factor intervention for early management, diagnosis and treatment of early disease are important in stemming the epidemic of cardiovascular disease in this population, attention also needs to be paid to factors associated with ethnicity.

## Competing interests

The authors declare that they have no competing interests.

## Authors' contributions

BAH MSc performed the cardiovascular ultrasounds, collected the clinical data, analysed the data and wrote the original manuscript draft. LC MD recruited the patients and collected clinical data. LJ BS assisted in performing the ultrasounds and collecting data. AAS, MA MSc MD recruited the patients and collected clinical data. JS MD PhD provided help in study design and analysis. THM MD PhD provided help in study design, technical mentoring, interpreting data and edited the final manuscript draft.
